# DeepSub: Utilizing Deep Learning for Predicting the Number of Subunits in Homo-Oligomeric Protein Complexes

**DOI:** 10.3390/ijms25094803

**Published:** 2024-04-28

**Authors:** Rui Deng, Ke Wu, Jiawei Lin, Dehang Wang, Yuanyuan Huang, Yang Li, Zhenkun Shi, Zihan Zhang, Zhiwen Wang, Zhitao Mao, Xiaoping Liao, Hongwu Ma

**Affiliations:** 1 College of Biotechnology, Tianjin University of Science and Technology, Tianjin 300457, China; 2 Haihe Laboratory of Synthetic Biology, Tianjin 300308, China; 3 Biodesign Center, Key Laboratory of Engineering Biology for Low-Carbon Manufacturing, Tianjin Institute of Industrial Biotechnology, Chinese Academy of Sciences, Tianjin 300308, China; 4 Institute of Biopharmaceutical and Health Engineering, Tsinghua Shenzhen International Graduate School, Tsinghua University, Shenzhen 518055, China; 5 University of Chinese Academy of Sciences, Beijing 100049, China; 6 School of Computer Science and Technology, Beijing Institute of Technology, Beijing 100081, China; 7 Key Laboratory of Systems Bioengineering (Ministry of Education), Frontier Science Center for Synthetic Biology (Ministry of Education), Department of Biochemical Engineering, School of Chemical Engineering and Technology, Tianjin University, Tianjin 300072, China

**Keywords:** homo-oligomers, subunit, deep learning, protein language model

## Abstract

The molecular weight (MW) of an enzyme is a critical parameter in enzyme-constrained models (ecModels). It is determined by two factors: the presence of subunits and the abundance of each subunit. Although the number of subunits (NS) can potentially be obtained from UniProt, this information is not readily available for most proteins. In this study, we addressed this gap by extracting and curating subunit information from the UniProt database to establish a robust benchmark dataset. Subsequently, we propose a novel model named DeepSub, which leverages the protein language model and Bi-directional Gated Recurrent Unit (GRU), to predict NS in homo-oligomers solely based on protein sequences. DeepSub demonstrates remarkable accuracy, achieving an accuracy rate as high as 0.967, surpassing the performance of QUEEN. To validate the effectiveness of DeepSub, we performed predictions for protein homo-oligomers that have been reported in the literature but are not documented in the UniProt database. Examples include homoserine dehydrogenase from *Corynebacterium glutamicum*, Matrilin-4 from *Mus musculus* and *Homo sapiens*, and the Multimerins protein family from *M. musculus* and *H. sapiens*. The predicted results align closely with the reported findings in the literature, underscoring the reliability and utility of DeepSub.

## 1. Introduction

Protein oligomerization is a well-established phenomenon responsible for their functionality in biological systems, encompassing eukaryotic and prokaryotic organisms, involving approximately 30% of total proteins [1]. Protein oligomers have recently garnered significant interest in the fields of structural biology [2], chemical biology [3], and neurodegeneration [4]. These oligomers usually consist of a limited number of subunits (NS), ranging from two to ten, offering substantial combinatorial potential, particularly through both hetero-oligomerization and homo-oligomerization [5]. Furthermore, it is well-established that homo-oligomers play crucial roles in mediating and regulating processes such as gene expression [6], enzyme function [7], ion channels [8], receptors [9], and cell–cell adhesion [10]. Therefore, understanding the homo-oligomers is vital at the molecular level to comprehend the physiological functions of proteins and design molecular regulators for their modulation [11].

Furthermore, in ecModels, the turnover number (*k*_cat_) and MW of an enzyme impose constraints on the fluxes of reactions catalyzed by the enzyme, thereby crucially impacting the predictive accuracy of ecModels. In conventional enzyme-constrained modeling frameworks like GECKO [12], enzymes are typically assumed to function as monomers, with their MW derived solely from the amino acid sequence, which deviates from reality. ECMpy and ETGEMs utilize curated information from the UniProt [13] database regarding protein subunit composition to accurately assign NS to a subset of proteins within the model, such as dimers or tetramers [14,15]. However, the descriptions of protein subunit composition in the UniProt database are limited, not comprehensive across all species, and, even for model organisms, they lack complete coverage. While an increasing number of studies have focused on developing artificial intelligence methods (such as DLKcat [16], TurNup [17], UniKP [18], etc.) to predict *k*_cat_ and enhance its coverage in models, relatively few have addressed MW.

Two primary factors influence the final MW assigned to an enzyme involved in a specific reaction: whether the protein consists of subunits (as indicated by an “and” relationship in the gene–protein reaction, or GPR, associations) and the abundance of each subunit. Although obtaining the MW of a protein may seem straightforward through databases like UniProt or computational methods based on protein sequences, the MW values obtained from these sources typically represent monomers. For instance, 6-phosphogluconate dehydrogenase encoded by gene b2029 in *Escherichia coli* is a homodimer, resulting in an MW of 102.962 kDa rather than 51.481 kDa. Additionally, many enzymes comprise subunits encoded by different genes, denoted by “and” relationships in genome-scale metabolic models (GEMs). However, the GPR relationships often lack information regarding the number of each subunit in the protein complex. For example, Succinyl-CoA synthetase is a heterotetramer containing two alpha subunits (encoded by b0729 with an MW of 29.777 kDa) and two beta subunits (encoded by b0728 with an MW of 41.393 kDa). Consequently, the MW of this enzyme complex should be 142.34 kDa instead of 71.17 kDa. Although the number of each subunit can potentially be retrieved from UniProt, this information is missing for many proteins. Using *E. coli* as an example, there are currently 902 proteins with clear homo-oligomeric states in the Swiss-Prot database, among which only 238 are monomers, with the remainder being oligomers. As a result, obtaining quantitative information on enzyme subunit composition is challenging, often leading to incorrect MW values in published ecModels, which, in turn, affects the prediction accuracy of ecModels.

Experimental approaches are typically employed to determine the NS of proteins, such as X-ray and neutron scattering, mass spectrometry, size exclusion chromatography, gel filtration, dynamic light scattering, analytical ultracentrifugation, and fluorescence resonance energy transfer [19]. While effective, these methods can be costly and labor-intensive. To address these challenges, computational protocols have emerged, often leveraging solved crystal structures as a starting point. However, these methods have limitations, particularly in cases where experimental structures are unavailable [20]. Recent advancements in deep learning have shown promise in predicting protein’s quaternary state. Protein language models, utilizing computational natural language processing techniques for proteins, have successfully captured secondary structure, protein cellular localization, and other features from amino acid sequences [21]. This raises the question: can a protein’s quaternary state be inferred solely from its sequence? Orly et al. introduced “QUEEN” [21], a study exploring the use of the pretrained model Evolutionary Scale Modeling 2 (ESM2) [22] for predicting protein quaternary state from sequences. However, we have observed over 50% of protein fragments in the training dataset. Consequently, employing QUEEN for direct prediction of homo-oligomeric states for whole-length protein might yield unreliable outcomes.

In this research, we present a new model named DeepSub for NS prediction based on the protein language model and Bi-directional GRU. We compared the performance of DeepSub with QUEEN and found that DeepSub consistently outperforms the latter, achieving an accuracy rate of 96.7%. Furthermore, case studies show that DeepSub can successfully predict subunit structure for proteins not included in the UniProt database.

## 2. Results and Discussion

### 2.1. Processing and Analyzing Datasets Extracted from UniProt

**Figure 1:**
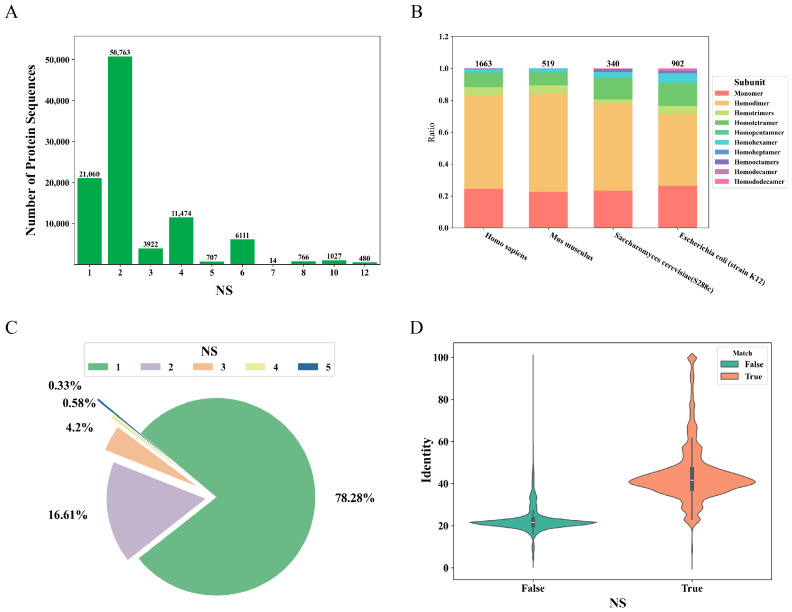
Analysis of homo-oligomer dataset from Swiss-Prot. (**A**) Distribution of the NS. (**B**) Distribution of monomers and homo-oligomers for four model organisms. (**C**) Distribution of NS for proteins with the same EC number. (**D**) Sequence similarity distribution for protein pairs with the same EC number when NS is the same or different.

Initially, we extracted 570,420 entries from the UniProt (Swiss-Prot) database. After removing 278,953 entries without subunit descriptions and filtering out 71,191 entries described by sequence similarity (ECO: 0000250), we processed the remaining 220,276 entries using specific keywords (Table S1) and obtained 101,801 entries. Next, entries with descriptions containing “By similarity”, “Probable”, or “Potential” were filtered out, resulting in a dataset comprising 96,324 entries (https://github.com/tibbdc/DeepSub/tree/main/DATA, accessed on 26 March 2024). An analysis of the label distribution within the dataset unveiled a significantly elevated proportion of homodimers and monomers compared to other homo-oligomers. Overall, even-numbered homo-oligomers were more prevalent than odd-numbered homo-oligomers (Figure 1A). Furthermore, we performed a species-specific analysis on four extensively studied organisms with the collected data and found that the proportion of proteins with a precise homo-oligomer state available is relatively low, with the highest being 20% in *Escherichia coli* (Figure 1B). Homodimers are found most prevalent across the four species, with proportions of 58.45%, 61.66%, 54.71%, and 45.01% in *Homo sapiens*, *Mus musculus*, *Saccharomyces cerevisiae* (S288c), and *E. coli* (strain K12), respectively. *E. coli* exhibits the richest diversity in multimers, but *H. sapiens* lacks homododecamer, *M. musculus* lacks homooctamers, homodecamer, and homododecamer, and *S. cerevisiae* lacks homodecamer and homoheptamer (Table S2).

In addition, we investigated the distribution of NS for proteins with the same EC number (a total of 2575 EC numbers). Among them, 78.28% of the EC numbers were associated with only one NS, while 16.61% of the EC numbers had two NS (Figure 1D). Interestingly, we found that there are nine EC numbers with the most diverse homo-oligomer states, namely 1.15.1.1, 2.5.1.41, 2.7.7.7, 3.2.1.21, 3.4.11.5, 3.5.1.4, 3.6.1.1, 3.6.1.15, and 4.2.1.1 (Table 1). These proteins have the same EC number but different NS, reflecting their diversity in multimeric structures. Among these nine EC numbers, the enzymes with the highest counts are 1.15.1.1 superoxide dismutase, 2.7.7.7 DNA-directed DNA polymerase, and 3.6.1.1 inorganic diphosphatase. The evolutionary histories of these enzymes are notably extensive, indicating the possible emergence of diverse homo-oligomeric states throughout prolonged evolutionary processes (Figure 1C).

Moreover, we paired proteins with the same EC number and conducted pairwise sequence alignment. As shown in Figure 1D, it can be observed that, when NS is the same (label_match is True), the overall similarity is higher than the case when NS is different. However, a considerable proportion of protein pairs with the same NS exhibit less than 30% sequence similarity. At the same time, there is also a considerable proportion of proteins with over 30% similarity but different NS. So, relying solely on sequence similarity (e.g., a threshold of 30%) to predict the oligomeric state of proteins may not always be reliable, a point that has been mentioned in earlier research [21] as well.

### 2.2. Cross-Validation on the Training Set

To comprehensively assess the performance of the DeepSub model, we employed the 10-fold cross-validation. The entire training set is randomly divided into 10 parts. In each round, nine parts are used for training while the rest is used for testing. As shown in Table 2, the 10-fold cross-validation demonstrates that DeepSub’s prediction performance is exceptionally good, with minimal fluctuations. The average macro-accuracy reached 97%; additionally, there was a macro-recall rate of 0.897 and a macro-F1 score of 0.905. These metrics collectively demonstrate the model’s superior prediction accuracy and stability. Subsequently, we evaluated the performance of DeepSub in predicting subunit categories and found that it failed to predict heptamers accurately. This is mainly attributed to the scarcity of heptamer samples in our dataset, comprising only 14 instances. The small sample size severely limits the model’s ability to perform well in this category and impacts its overall predictive accuracy. This underscores the necessity in future studies to augment the sample size for individual subunit categories, thereby enhancing the model’s generalization capabilities and improving prediction accuracy.

### 2.3. Comparison with QUEEN

**Figure 2:**
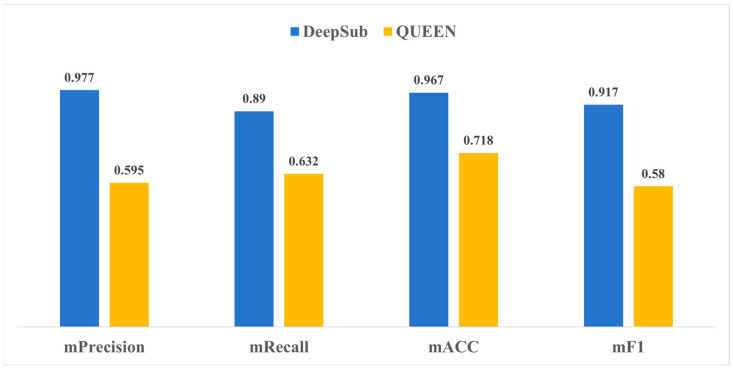
Performance comparisons between DeepSub and QUEEN.

We conducted comparisons with the deep learning method QUEEN. The test results, as shown in Figure 2, demonstrate the exceptional overall performance of DeepSub. The mACC of DeepSub reached 0.967, significantly outperforming QUEEN, which attained a score of only 0.718 (Figure 2). Furthermore, DeepSub demonstrated superior performance in terms of mRecall, with a score of 0.890, indicating its strong capability in correctly identifying positive cases of subunits (Figure 2). The model also achieved high scores in precision and mF1 score as well, with 0.977 and 0.917, respectively (Figure 2). These metrics collectively reflect the high accuracy of the DeepSub model in NS prediction. In addition, the results on the test dataset are highly consistent with those of the 10-fold cross-validation, demonstrating the excellent generalization ability of the DeepSub model.

### 2.4. Case Study

Furthermore, we conducted predictions for protein oligomers that were reported in the literature but not recorded in the UniProt database. Firstly, crystal structures of homoserine dehydrogenase (HSDs), which plays a pivotal role in the aspartate pathway [23], from multiple microbial sources have been elucidated, revealing a catalytic mechanism wherein the enzyme exists as either a dimer or a tetramer [24,25]. However, the crystal structure of CgHSD (P08499, the HSD of *Corynebacterium glutamicum*) remains unreported, and the subunit structure is absent from UniProt databases. DeepSub predicts CgHSD to be a homotetramer, consistent with prior research confirming its oligomeric state via size-exclusion chromatography (SEC) [26]. However, QUEEN incorrectly predicts it as a homodimer in this particular example (Table 3).

**Figure 3:**
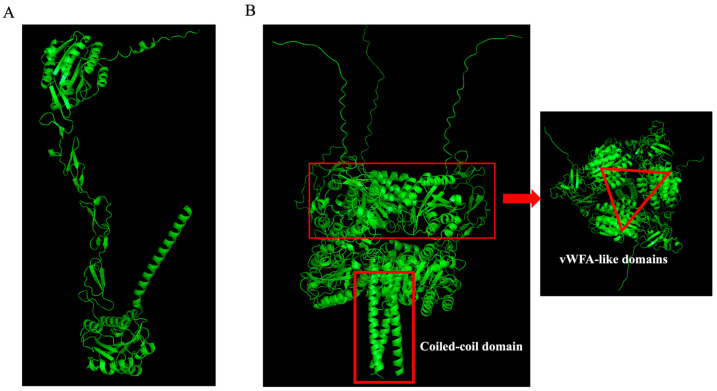
The monomeric (**A**) and trimeric (**B**) structures of protein O89029 predicted by AlphaFold-Multimer.

Furthermore, Matrilin-4 represents the most recently identified member of the matrilin family, characterized by von Willebrand factor-A-like domains and serving as extracellular matrix adapter proteins [27]. DeepSub predicts that Matrilin-4 in *Mus musculus* and *Homo sapiens* form homotrimeric structures, while QUEEN predicts it to be a monomer (Table 3). A previous study has demonstrated that, in *M. musculus*, SDS-PAGE analysis, MALDI-TOF mass spectrometry, and electron microscopy confirmed the production of Matrilin-4 homotrimers in 293-EBNA cells transfected with Matrilin-4 cDNA [28]. Electron microscopy revealed that the trimeric form exhibits similarities to the bouquet-like shape observed in other matrilins, featuring a compact center from which stalk-like structures with globular ends extend [28]. These findings validate the accuracy of DeepSub’s predictions. Subsequently, we utilized AlphaFold-Multimer [29] to predict the protein structure of the Matrilin-4 in *M. musculus*. The prediction unveiled a trimeric structure of the complex, exhibiting resemblances to a bouquet-like shape (Figure 3B). This structure encompasses the C-terminal coiled-coil domains and the N-terminal vWFA-like domains (Figure 3A,B), aligning with previously documented observations in electron microscopy [28].

Lastly, Multimerins, comprising Multimerin-1 and Multimerin-2, form a two-member family characterized by a shared C-terminal globular domain of C1q (gC1q) domain typical of the gC1q/TNF superfamily, alongside a unique N-terminus cysteine-rich EMI domain [30]. Multimerin-1, a large, soluble, disulfide-linked homopolymeric protein, is expressed in megakaryocytes, platelets, and endothelial cells [31]. On the other hand, Multimerin-2, an extracellular matrix glycoprotein, has an elusive function, although Marastoni et al. observed its significant impact on endothelial cell (EC) migration and the organization of a functional vessel network [32]. The crystal structure of Multimerins in *M. musculus* and *H. sapiens* remains unreported, and subunit structure information is absent from UniProt databases. While DeepSub predicts that Multimerins in both species form trimers, the alternative method, QUEEN, suggests dimers (Table 3). Verdone et al. pioneered the determination of the three-dimensional NMR solution structure of the human EMILIN1 gC1q homotrimer [33], revealing striking homology to the gC1q domains of several other members of the C1q/TNF superfamily. Furthermore, we conducted structure alignment [34] between the trimeric structure predicted and the human EMILIN1 gC1q homotrimer. The result showed a TM-score of 0.586, indicating the presence of a gC1q homotrimer in the predicted trimer.

### 2.5. Web Platform

**Figure 4:**
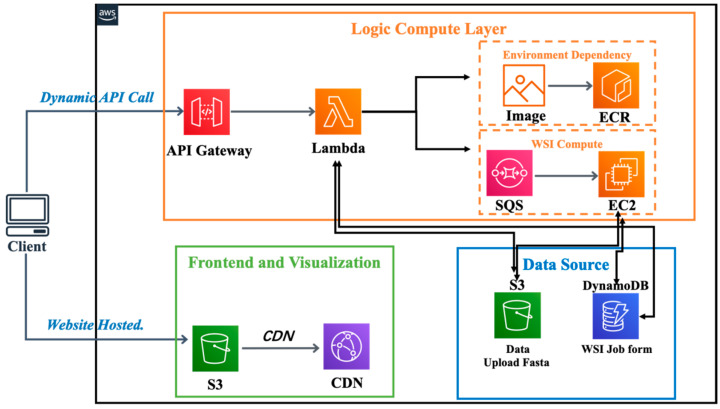
The architecture of DeepSub. A three-tier architecture: front presentation, logic computation, and data storage tiers. Data, including user files, is managed by AWS DynamoDB and S3. The front tier is hosted on S3 and accelerated by CloudFront, while the logic tier handles computation.

DeepSub was built entirely on cloud-based architecture (Figure 4). We used a three-tier architecture (the front presentation tier, logic computation tier, and data storage tier) to build our web server on Amazon Web Services. The data storage tier manages the persistent storage of our platform, including AWS DynamoDB and AWS S3, which store user-uploaded input files, parameters, and jobs. The front presentation tier represents the components users directly interact with, which is hosted by the AWS S3 static website functionality and accelerated by AWS CloudFront. The logic computation tier manages requests from external systems and performs the prediction.

## 3. Materials and Methods

### 3.1. Datasets

We have observed discrepancies between the tabular data and the web-based data provided by UniProt, particularly in cases where the NS of a protein is inferred through similarity calculations. These evidence data are not displayed in the tabular format. Therefore, we have adopted an alternative approach: directly parse XML files to retrieve data. We used Python’s XML parsing library to handle data from UniProt, specifically extracting detailed biological information about proteins to generate a structured dataset, such as the UniProt ID for each protein entry, descriptions of protein NS, protein sequences, EC numbers, as well as evidence types, and specific subunit evidence related to NS. To handle large-scale XML files efficiently, we utilized the iterparse method from the lxml library. This approach alleviates the memory burden when parsing each entry element and maintains high processing efficiency. During parsing, relevant information of each entry is precisely extracted and structured into a dictionary, which is then appended to an accumulating data list. Furthermore, we promptly clear related XML elements after processing each entry to optimize memory usage further. This method ensures the accuracy and integrity of data processing while guaranteeing efficiency and optimized memory usage throughout the process.

We removed proteins that lacked descriptions of subunits and filtered the subunit evidence indicating NS using sequence similarity in manual assertions, denoted in UniProt by Term ID (ECO:0000250). Then, we observed that NS of proteins are described using terms like monomer or homodimer and extracted data containing the following 10 subunit labels: monomer, homodimer, homotrimer, homotetramer, homopentamer, homohexamer, homoheptamer, homooctamer, homodimer, and homo-dodecamer. However, multiple types of subunit labels may appear in the description. For example, the UniProt entry “O15537” corresponds to the retinoschisin in *Homo sapiens*. In its “interaction” term, it is described as “Homooctamer of 4 homodimers; disulfide-linked (PubMed:15644328, PubMed:19849666). The homooctamer has a flat, cogwheel structure with a diameter of about 14 nm (PubMed:27798099, PubMed:26812435, PubMed:27114531). Two stacked octamers can assemble to form a hexadecamer (PubMed:27798099, PubMed:26812435, PubMed:27114531)”. Labels assigned based solely on the subunit labels “homooctamer” and “homodimers” would be incorrect for this protein. To reduce the number of false positive samples in the dataset, we conducted a systematic review of the “interaction” terms in the UniProt and summarized a mapping between keywords and NS as a criterion for data screening (Table S1). This mapping is used to filter data based on specific keywords and assign labels. For instance, by using the specific keyword “Homooctamer of”, the retinoschisin (O15537) would be assigned as “8”. To ensure data reliability, we only matched data from Swiss-Prot, the reviewed items in UniProt.

### 3.2. The model Architecture of DeepSub

**Figure 5:**
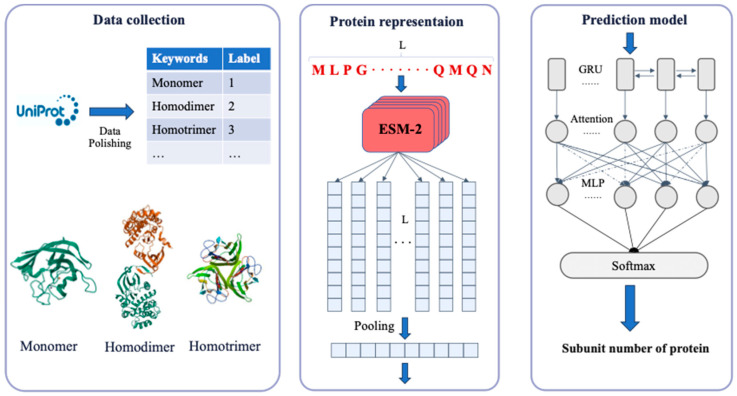
The model architecture of DeepSub. The process involves three main steps: downloading and cleaning data from the Swiss-Prot database, embedding proteins using the ESM2 protein language model, and utilizing GRU and attention mechanisms for NS prediction.

The model architecture of DeepSub is shown in Figure 5. Firstly, semantic representations of protein sequences are obtained through ESM2 [22], which is an advanced protein language model aimed at understanding and predicting the structure and function of proteins [35]. Subsequently, the downstream NS prediction tasks are learned using an architecture based on a Bi-directional GRU and an attention layer [36]. Lastly, the output from the attention layer is connected to a fully connected output layer with a Softmax activation function corresponding to the number of categories, resulting in the probability of each category. In our study, we first represent the input protein sequence P(resi1,resi2,...,resil), where the length of the protein sequence is *l*. Next, we apply the ESM2 model to embed the protein sequence, resulting in a matrix of dimensions l × *n*, where *n* = 1280 represents the embedding dimension of the protein. To further process this matrix, we perform a pooling operation to compress its dimensions from l×1280 to 1×1280. This processed matrix is then used as the input for the Bi-directional GRU layer, which contains 128 hidden units. The bi-directional structure of the GRU layer allows the model to capture sequence dependencies from both forward and backward directions, and this complete view of context often performs better in sequence tasks. This GRU component of this process can be represented as:

(1) $$H_{gru}=f_{gru}\left(x\right)$$

where $H{\;}_{gru}$ ∈ R^^(1×H)^ is the output from the GRU layer, is the function of the bidirectional GRU layer, and *x* ∈ R^^(1×1280)^ is the result of applying a pooling layer to the ESM2 embeddings of the input protein sequence.

The second key component of the model is an attention layer. This mechanism, widely used in deep learning, allows the model to give varying degrees of attention to each input element based on its importance. In our model, our attention layer is configured with 32 attention heads. This means the model can pay attention to multiple parts of the sequence simultaneously at each timestep, thus endowing the model with greater expressive capability. This process can be represented as:

(2) $$H\_attention=f\_attention(H_{gru})$$

where H_attention ∈ R^^(1×A)^ is the output from the attention layer, is the function of the attention layer, and $H_{gru}$ is the output from the previous Bi-directional GRU layer.

The final part of the model is a fully connected layer. It takes the output from the attention layer and transforms it into the model’s final prediction. Mathematically, this can be represented as:

(3) Y=f_fc(H_attention)

where Y is the final output of the model, f_fc is the function of the fully connected layer, and H_attention is the output from the previous attention layer. Therefore, the entire model can be represented by the following formula:

(4) $$Y=f\_fc(f\_attention(f_{gru}(X)))$$

The above model structure combines the temporal processing capability of Recurrent Neural Networks and the context attention capability of the attention mechanism, enabling us to effectively extract useful features from the input protein sequence representation and incorporate important contextual information into the final representation.

### 3.3. Model Training

We divided the dataset into training and testing sets in an 8:2 ratio. The design and construction of the DeepSub model exemplify the fusion of advanced technologies in contemporary bioinformatics and computational biology. Developed in Python 3.10, it integrates the biological data processing capabilities of Biopython 1.79 and operates efficiently within the cudatoolkit 11.8 environment. DeepSub incorporates TensorFlow 2.14.0 and PyTorch 2.2.0, two leading deep learning frameworks at its core. Trained on the NVIDIA GeForce GTX A6000 graphics card with 48 GB of memory, DeepSub benefits from powerful parallel computing and large-scale data handling capabilities. During training, the learning rate is set at 0.001, aimed at precise model optimization through gradual weight adjustments. The model undergoes 200 training epochs, ensuring ample time for learning and feature extraction from the data. A batch size 1024 accelerates training by processing large volumes of data in each iteration. A dropout rate of 0.5 is implemented to prevent overfitting, balancing complexity with improved generalization capabilities.

### 3.4. Baseline Models

The QUEEN model utilizes the Qsbio training dataset and employs the ESM-2 model for protein embedding as its foundation. The classification component is handled by a multilayer perceptron (MLP) configured with specific parameters. In this classifier, the activation function “identity” is used, meaning that the output layer directly outputs a result without any activation processing. However, the retraining code for the QUEEN model is not publicly available; the production model of QUEEN was used in this study.

### 3.5. Loss Function

In this task, we use a loss function that is commonly used for multi-classification problems. This function calculates the cross-entropy between the true distribution and the predicted distribution. The specific formula is as follows:

(5) H(p,q)=−Σp(x)logq(x)

where *p* is the true distribution, *q* is the predicted distribution, Σ is the summation over all categories, *x* is a specific category, *p*(*x*) is the probability of category *x* in the true distribution, and *q*(*x*) is the probability of category *x* in the predicted distribution.

### 3.6. Evaluation Metrics

To assess the performance of the model, four commonly used metrics were calculated and defined: *mACC* (macro-average accuracy), *mPrecision* (macro-average precision), *mRecall* (macro-average recall), and *mF1* (macro-average F1 score).

(6) $$mACC=\frac{\sum_{i=1}^{n}\frac{{TP}_{i}+{TN}_{i}}{{TP}_{i}+{TN}_{i}+{FP}_{i}+{FN}_{i}}}{n},n=1,2,3,\cdots ,N$$

(7) $$mPrecision=\frac{\sum_{i=1}^{n}\frac{{TP}_{i}}{{TP}_{i}+{FP}_{i}}}{n},n=1,2,3,\cdots ,N$$

(8) $$mRecall=\frac{\sum_{i=1}^{n}\frac{{TP}_{i}}{{TP}_{i}+{FN}_{i}}}{n},n=1,2,3,\cdots ,N$$

(9) $$mF1=\frac{2\times mPrecision\times mRecall}{mPrecision+mRecall}$$

where *TP_i_*, *TN_i_*, *FP_i_*, and *FN_i_* denote the numbers of true positive, true negative, false positive, and false negative samples for the *i*th class, respectively.

## 4. Conclusions

In traditional protein databases, information is typically organized around genes or proteins, including repositories of protein structures. However, in reality, homo-oligomers do not function as individual monomers, and their structures and characteristics vary significantly from those of monomers. AlphaFold-Multimer can predict the three-dimensional structure of protein complexes using amino acid sequences as input but requires the users to know the NS. For homo-oligomers, knowing the specific oligomeric state is essential when preparing the sequence file. However, in practical applications, researchers often encounter challenges in determining the actual NS of the target protein, which can hinder the attainment of optimal results. In this study, we obtained sequences and corresponding NS for monomeric and oligomeric proteins from UniProt. Leveraging deep learning techniques and the protein language model, we developed DeepSub to predict NS in homo-oligomers based solely on amino acid sequences, thereby aiding in the prediction of true protein structures.

Furthermore, the MW of an enzyme represents a critical parameter in ecModels. Obtaining quantitative information on enzyme subunit composition is challenging, often resulting in inaccurate MW values in published ecModels. The method proposed in this study for predicting protein NS can effectively address this limitation, which is crucial for enhancing the accuracy and reliability of ecModels.

Although our method demonstrates high precision in many aspects, it still faces certain limitations. Primarily, our data originate solely from the UniProt database, which currently offers an insufficient volume of data, particularly for samples like heptamers, which are limited to only 14 instances, as discussed in Section 2.2. This highlights the need for future studies to increase the sample size for specific subunit categories, thereby improving the model’s generalization capabilities and predictive accuracy. Additionally, there is room for improvement in the interpretability of our method. We will strive to introduce an interpretative mechanism into the model to identify the key residues influencing the conformation. This will not only enable us to more accurately understand the scientific principles behind the prediction results but also enhance the practicality of our method, laying a stronger foundation for future scientific research.

## Figures and Tables

**Table 1:** The EC numbers with the most diverse NS.

EC Number	Protein Name	Protein Counts	Subunits Labels
1.15.1.1	superoxide dismutase	126	1, 2, 3, 4, 6
2.5.1.41	phosphoglycerol geranylgeranyltransferase	11	1, 2, 4, 5, 6
2.7.7.7	DNA-directed DNA polymerase	217	1, 2, 3, 4, 6
3.2.1.21	beta-glucosidase	12	1, 2, 4, 6, 8
3.4.11.5	prolyl aminopeptidase	8	1, 2, 3, 4, 6
3.5.1.4	amidase	6	1, 2, 4, 6, 8
3.6.1.1	inorganic diphosphatase	116	1, 2, 3, 6, 12
3.6.1.15	nucleoside-triphosphate phosphatase	16	1, 2, 4, 6, 12
4.2.1.1	carbonic anhydrase	13	1, 2, 3, 4, 6

**Table 2:** Ten-fold cross-validation on the training set.

Fold	mPrecision	mRecall	mACC	mF1
1	0.882	0.861	0.970	0.871
2	0.946	0.958	0.970	0.948
3	0.976	0.916	0.970	0.937
4	0.883	0.857	0.970	0.870
5	0.977	0.9648	0.972	0.970
6	0.8795	0.870	0.971	0.875
7	0.876	0.862	0.970	0.869
8	0.879	0.856	0.968	0.867
9	0.980	0.964	0.969	0.971
10	0.880	0.864	0.969	0.871
Average	0.916 ± 0.047	0.897 ± 0.048	0.97 ± 0.001	0.905 ± 0.046

**Table 3:** NS prediction for cgHSD, Matrilin-4, Multimerin-1, and Multimerin-2.

Organism	Uniprot ID	Complex	QUEEN	DeepSub
*Corynebacterium glutamicum*	P08499	-	4	2
*Mus musculus*	O89029	Matrilin-4 complex	1	3
*Homo sapiens*	O95460	Matrilin-4 complex	1	3
*Mus musculus*	B2RPV6	Multimerin-1 complex	2	3
*Homo sapiens*	Q13201	Multimerin-1 complex	2	3
*Mus musculus*	A6H6E2	Multimerin-2 complex	2	3
*Homo sapiens*	Q9H8L6	Multimerin-2 complex	2	3

## Data Availability

The standalone version and related data are provided at https://github.com/tibbdc/deepsub (accessed on 26 March 2024). Moreover, we developed the web platform https://deepsub.biodesign.ac.cn/ (accessed on 26 March 2024) for the convenience of end-users.

## Supplementary Materials

The following supporting information can be downloaded at: https://www.mdpi.com/article/10.3390/ijms25094803/s1.
